# Editorial: Women's Health in Low-Resourced Countries: Epidemiology, Governance, Advocacy, Capacity

**DOI:** 10.3389/fpubh.2020.602467

**Published:** 2020-12-09

**Authors:** Ulrich Laaser, Vesna Bjegovic-Mikanovic, Motasem Hamdan, Flavia Senkubuge, Michaela Michel-Schuldt

**Affiliations:** ^1^Faculty of Health Sciences, Bielefeld University, Bielefeld, Germany; ^2^Institute of Social Medicine, University of Belgrade, Belgrade, Serbia; ^3^School of Public Health, Al-Quds University, Jerusalem, Palestine; ^4^School of Health Systems and Public Health, Faculty of Health Sciences, University of Pretoria, Pretoria, South Africa; ^5^EPOS Health Management, Bad Homburg, Germany

**Keywords:** health systems, mother and child health, South Eastern European countries, Africa, countries in transition

The starter for this Research Topic in Frontiers in Public Health and Public Health Policy was the recent experience of members of the editorial team in West-Africa. Women's health, especially maternal health, in most regions of the global south is part of the unfinished agenda as stated in the United Nation's Millennium Development Goals Report 2015, missing the set targets for Goal 5: Improve Maternal Health. As of 2015, some of the highest maternal mortality ratios were recorded in West Africa, e.g., as 1,360 per 100,000 live births in Sierra Leone or 814 in Nigeria (1) whereas Western countries usually stay between 5 and 10. Thus, it is legitimate to ask whether now the chances to reach SDG-3 in 2030 are better. We analyzed that, taking as an example Liberia—a small Anglophone coastal country in West-Africa. According to trends calculated on the basis of national survey data recorded in 2007 and 2013, Liberia is in delay by more than 10 years for maternal mortality and as well for neonatal mortality, in other words unlikely to achieve the SDG-3 target in time. However, the quality of data is a problem as WHO for example paints a less unfavorable picture based on estimates. A more encouraging example has been described and analyzed in the contribution from Ghana—one of the most advanced African countries. The study concludes that in a male dominated society the involvement of males into pregnancy and delivery—contrary to the inherited culture—makes a positive difference, especially in the selection of the place of delivery. Less in Ghana, more so in Liberia the most relevant upstream determinants of poor maternal health in the developing world are unreliable transport on roads, insufficient electricity, and lack of connectivity for online communication. On site, it includes lack of qualified and motivated, often underpaid health staff and regulated continuing professional training. Similarly technical equipment, its maintenance, and regularly controlled stocks of essential drugs are rarely satisfying, at least in the remote settlements where large parts of the population lives.

In Europe, we are confronted with other burdening problem sets. Two papers deal with the quite large Roma populations in South Eastern Europe. In the Republic of Srpska—in spite of health insurance and universal health access are legally guaranteed—we find Roma women that had less education, were unemployed, divorced or widowed, have unmet health needs and lack adequate medical supervision of a chronic condition. A second study compares desirable exclusive breastfeeding in the Roma and non-Roma Serbian population. Habits differ considerably but most striking is the observation that not a single Roma woman attended a childbirth preparation program. In summary the statement seems to be justified that also in Europe we can observe female populations disadvantaged by origin. Even more disturbing that one third of women worldwide experience at some moment in their lives physical violence even from their intimate partners. In North Macedonia Violence against women is the most common form of human rights violation against women and it has a negative impact on health, causing injuries, death, as well as psychological trauma. Again, minority groups, especially the Roma population are exposed the most.

How can that be solved, to realize the guarantied rights to all genders instead of careful analyses leading to inefficient declarations. Two papers from Albania and from Greece call for women leadership. The Albanian study shows progress in female leadership qualification during the last decade however leaving behind elderly women. The Greek paper calls Gender inequalities an important derailment factor for health workforce and health system sustainability. It identifies a gap in the evidence based research on women leaders' own perceptions of barriers, especially work/life balance, lack of equal career advancement, and lack of confidence, in total 26 marked barriers, a rich array for further research.

It is the hope of the editorial group that this collection initiates more exploration of the concrete obstacles hindering females in low-resourced countries to contribute their—in many ways lost—potential to improve the world's well-being: for example, given the importance of the issue, by organizing midwifery in their own professional chambers, not the case even in all advanced countries. The editors received publishable contributions from two regions, West-Africa and South Eastern Europe, but there are restrictions and even iron barriers for full female participation also in advanced social environments. The high maternal and newborn mortality in the global South and the expected failure to reach the SDG3 targets in 2030 could be enhanced considerably by strengthening an equally footed female contribution. There is an abundance of actors, statistics, and financial support systems but there is still lack of confidence that women can take their fate into own hands. The contributions in this issue, however, demonstrate that women are on the march!

## Author Contributions

The editorial has been written by UL and approved by the co-editors.

## Conflict of Interest

The authors declare that the research was conducted in the absence of any commercial or financial relationships that could be construed as a potential conflict of interest.

